# MENTAL AND PHYSICAL HEALTH EFFECTS OF EVERYDAY DISCRIMINATION TRAJECTORIES

**DOI:** 10.1093/geroni/igz038.2820

**Published:** 2019-11-08

**Authors:** Collin Mueller, Carlos Tavares

**Affiliations:** 1 Duke University, Durham, North Carolina, United States; 2 Lafayette College, Easton, Pennsylvania, United States

## Abstract

How do perceived discrimination trajectories impact health of Black, Latinx, and White adults ages 50 and older? Few researchers have sought to discern between the health effects of perceived discrimination measured cross-sectionally versus longitudinally. We aim to address this gap by leveraging newly available 3-wave panel data. We analyze 3 waves of data from the Health and Retirement Study (HRS) (n=2830 individuals ranging in age from 50 to 84, observed from 2008-2016). We first estimate group-based trajectories of everyday discrimination using finite mixture models. Second, we use multinomial logistic regression to estimate the likelihood of group membership in one of the identified trajectories of everyday discrimination based on psychosocial and demographic covariates. Third, we estimate the effects of group membership in one of these trajectories on four health outcomes: functional mobility, self-rated health, a count of chronic health conditions, and depressive symptoms. We identify three latent group-based trajectories of perceived discrimination. Black Americans are more likely than Latinx or White Americans to be members of latent trajectories characterized by higher levels of perceived discrimination over time. Membership in higher-discrimination groups over time is associated with worse physical and mental health profiles. Including measures of trajectory membership fully mediates the relationship between cross-sectional perceived discrimination and chronic conditions. Levels of discrimination decline across cohorts, suggesting that younger individuals perceive higher levels of everyday discrimination than their older counterparts.

